# Weight control in older adults with knee osteoarthritis: a qualitative study

**DOI:** 10.1186/s12891-020-03480-2

**Published:** 2020-08-02

**Authors:** Wen-Ling Yeh, Yun-Fang Tsai, Kuo-Yao Hsu, Dave Weichih Chen, Jong-Shyan Wang, Ching-Yen Chen

**Affiliations:** 1 Department of Traumatology Orthopedics, Chang Gung Memorial Hospital at Linkou, Tao-Yuan, Taiwan; 2 College of Medicine, Chang Gung University, Tao-Yuan, Taiwan; 3 School of Nursing, College of Medicine, Chang Gung University, Tao-Yuan, Taiwan; 4 Department of Nursing, Gung University of Science and Technology, Tao-Yuan, Taiwan; 5 Department of Psychiatry, Chang Gung Memorial Hospital at Keelung, Keelung, Taiwan; 6 Department of Orthopedic Surgery, Chang Gung Memorial Hospital at Linkou, Tao-Yuan, Taiwan; 7 Division of Joint Reconstruction, Department of Orthopedic Surgery, Chang Gung Memorial Hospital at Keelung, Keelung, Taiwan; 8 Healthy Aging Research Center, Graduate Institute of Rehabilitation Science, Chang Gung University, Tao-Yuan, Taiwan; 9 Heart Failure Center, Department of Physical Medicine and Rehabilitation, Chang Gung Memorial Hospital at Keelung, Keelung, Taiwan

**Keywords:** Knee osteoarthritis, Older adults, Weight control, Perception

## Abstract

**Background:**

Knee osteoarthritis (OA) affects mostly older adults and its primary risk factor is obesity. This study sought to understand weight-control strategies, facilitators of and barriers toward weight control in older adults with knee OA who preferred not to undergo physician-recommended total knee arthroplasty.

**Methods:**

For this qualitative descriptive study, older outpatients (*N* = 118) were recruited from orthopedic clinics at three hospitals. Data were collected through face-to face, individual in-depth interviews using a semi-structured interview guide and analyzed using content analysis.

**Results:**

Among participants, only 25.4% had body weight in the normal range and 55.9% reported having controlled their weight. Their most common weight-control strategies were to control diet and to exercise and control diet together. Weight control was facilitated by desiring good health, wanting to improve walking or movement, perceiving that they had gained weight, wanting to look good, and advice from healthcare providers. Common barriers to participants’ weight control were perceiving that dietary control was not needed, controlling appetite was difficult, dietary control was difficult, and not eating was physically uncomfortable.

**Conclusions:**

Our findings help healthcare providers understand how older adults with knee OA perceive weight control and serve as a reference for developing weight-control programs. Health care providers can integrate these identified facilitators and barriers into a weight-control intervention program. The importance of weighing oneself every day, the meaning of body mass index, consulting with a dietician regularly to control weight, and providing appropriate knowledge about aging and weight control should also be included in any weight-control intervention program.

## Background

Osteoarthritis (OA), which is a common joint disorder in the elderly [1], was the 12th leading cause of years lived with disability in 2016 [2]. Knee OA is a major cause of joint pain and problems in daily functioning. These patients’ knee pain and physical functioning have been predicted to deteriorate based on their knee characteristics, clinical factors, and psychosocial factors [3]. Among these factors, obesity is the primary risk factor for knee OA [4, 5]. Indeed, people clinically defined as obese (body mass index [BMI] > 30 kg/m^2^) were four times more likely to have knee OA than those with BMI in a desirable range (≤25 kg/m^2^) [6]. The degree of obesity in early life was also associated with the risk of developing knee OA later in life [7]. Given population aging and the increasing prevalence of obesity [6], the trend of increasing knee OA prevalence is expected to continue.

Overweight or obese adults with knee OA who participated in behavioral weight-loss interventions in a systematic review and meta-analysis of randomized controlled trials showed moderate improvements in pain and physical function [8]. In other words, disability improved significantly when weight loss was > 5.1%, or a reduction rate of 0.24% per week [8]. Moreover, all obese/overweight people with OA, ≥ 45 years old, with activity-related joint pain, and without morning joint-related stiffness or morning stiffness lasting < 30 min are recommended to receive weight-loss treatment [9].

However, such people find weight loss difficult to achieve and maintain [10]. Few studies have addressed the barriers to weight control among patients with knee OA [11–13]. For example, most obese knee-OA outpatients (89%) surveyed in the UK had tried to lose weight by changing their diet, trying to exercise more, and joining a support group [11]. Barriers to losing the desired amount of weight were lack of motivation, knee-joint pain, pain in other joints, and lack of time [11]. Similarly, overweight, older Thai adults (*N* = 40) with knee OA who participated in a quadriceps-exercise and weight-management intervention reported not adhering to the weight-management component 6 and 12 months after it ended were mainly family members’ and individual eating habits, problems with calorie counts and food-intake record, not preparing meals by oneself, insufficient time to eat, and diet-related health problems [12]. Twenty US patients were interviewed 3 months before (*n* = 11) or after (*n* = 9) knee-replacement surgery regarding their barriers to healthy eating. Their commonly reported barriers were desiring high-fat/high-calorie foods, difficulty managing overeating at restaurants and evening snacks, and difficulty managing negative mood, e.g., stress, depression, boredom [13]. Among these studies, the only common barrier was lack of time.

Those with OA and joint symptoms that greatly affect their quality of life (QOL) and cannot be relieved by non-surgical treatment should consider joint-replacement surgery [9, 14]. However, surgeries for total joint replacement have long waiting lists in the UK [15], which means that these patients need to bear with their painful joint symptoms and live with lower QOL until the surgery. Moreover, evidence indicates that receiving joint-replacement surgery may not relieve all symptoms for some OA patients [16, 17], and 18% of patients are discontented with the outcome [18]. These contradictory evidence-based results may make it difficult for patients with knee OA to decide what treatment to choose.

Those who do not feel comfortable undergoing total knee arthroplasty (TKA) are challenged by how to relieve pain as well as maintain physical function and QOL. In the past 5 years, many intervention programs for this population have focused on exercise alone [19] or combined exercise and weight control [20–22]. Since diet is influenced by culture and preferences [12], more studies are needed, particularly in Asian cultures, to explore weight control in older adults with knee OA. Understanding this population’s perceptions of weight control can help healthcare providers provide suitable information to help them control their weight. Therefore, this study was undertaken to understand the weight-control strategies, facilitators of and barriers to weight control among older Taiwanese adults with knee OA who did not feel comfortable undergoing physician-recommended TKA.

## Methods

### Design

We used a qualitative descriptive design for this study, which was part of a large research series on older people with OA to maintain their physical function and QOL if they did not feel comfortable undergoing physician-recommended TKA. A qualitative design was selected to better understand older adults’ perceptions of weight control. Part of this work has been presented at the 27th European Congress of Psychiatry [23].

### Sample and setting

We recruited older adults by convenience from one medical center and two regional hospitals in northwestern Taiwan. Physicians or nurses in outpatient orthopedic surgical clinics were asked to refer older adult patients with knee OA. Referred cases were included in the study by these criteria: 1) diagnosed with knee OA, 2) ≥ 55 years old, 3) did not feel comfortable undergoing physician-recommended TKA, and 4) able to understand and speak in Mandarin Chinese. Outpatients were excluded for other comorbid conditions causing disability (e.g., stroke, gout, and rheumatoid arthritis).

### Data collection

Participants’ data were collected in one-on-one, face-to-face, in-depth interviews using an interview guide (Table 1). The interview guide was developed by the research team and pilot tested in two older adult patients with knee OA. As a result of the participants’ responses, no modification was made. Only the results of questions 7–9 were analyzed in this study. Interviews were audiotaped with participants’ permission. Before interviews, participants’ information was collected using a researcher-designed form for demographics (age, BMI, gender, education level, religious beliefs, marital status, number of children, and work status) and clinical information (time since osteoarthritis diagnosis, site of OA, current treatment for OA, and experience of undergoing TKA).

**Table 1:** Interview Guide

1. Please describe your understanding of OA (e.g., causes, treatment, management, progression).	
2. Based on current UK guidelines, people over age 45 with OA are encouraged to exercise. Do you take any exercise? If so, what type, how long, and how often?	
3. What do you think about exercising?	
4. What factors would facilitate and hinder your exercising?	
5. Do you intend to exercise at home?	
6. What kinds of needs/expectations do you have for home-based exercise training?	
7. Please tell me about your eating habits in a typical day/week.	
8. Have you ever controlled your weight? How did you do?	
9. What factors would facilitate and hinder your weight control?	

The research assistant (RA) who collected the data was an experienced, bachelor-prepared female nurse who was trained by working on our OA projects over 7 years. She collected data in audiotaped individual interviews, each lasting 20–60 min. The RA had no contact with eligible participants before recruitment. Participants were interviewed in Mandarin Chinese in a quiet room at participating hospitals; most participants were accompanied by their family. Immediately after each interview, The RA took field notes immediately after each interview to record observations about participants’ emotions or behaviors during interviews. Data collection proceeded until analysis showed no new themes (data saturation) for each selected hospital.

### Ethical considerations

This study was approved by the Institutional Review Board (IRB) of Chang Gung Medical Foundation (201601593B0), and the processes followed were in accordance with the ethical standards of the IRB and with the Helsinki Declaration of 1975, as revised in 1983. After IRB approval, physicians or nurses in outpatient orthopedic surgical clinics referred older adult patients with knee OA. The RA approached referred cases in each hospital’s orthopedic department waiting room and determined if they met the study criteria. The RA described the study purpose, procedure, interview time, confidentiality and rights to refuse participation or to withdraw from the study at any time. After older outpatients with OA decided to participate, they signed a consent form. A gift about 5 US dollars was provided after completing the interview to thank participants for their time.

### Data analysis

All audiotapes were transcribed verbatim within 48 h after interviews. Transcripts were analyzed by concept analysis [24]. First, the entire transcript and field notes for each participant were read and then reread several times to obtain a complete sense of the data. Next, all transcripts were analyzed to select and highlight significant phrases and statements. Data were then coded line by line. Finally, coded data were compared across transcripts to organize similar elements of experience, to identify emergent themes, and then frequency of each theme was counted. Before the study, the team had decided that each theme would be supported by at least 5% of the participants. Participants’ demographic and clinical information were analyzed by descriptive statistics (frequencies, percentages, means, and standard deviations) using a computer for analysis with SPSS/PC for Window 23.0 statistical software.

### Rigor

We established data trustworthiness by our extended engagement with transcripts, observing participants, peer debriefing, member checking and field notes [25]. For peer debriefing, the authors discussed the qualitative analysis with experts. For member checking, we asked three participants to review and check the validity of our findings. To ensure confirmability, the second author kept memos on decisions made throughout the study, thus allowing audit.

## Results

### Characteristics of participants

Of 131 older knee OA outpatients who met the study criteria, 11 declined to participate because of time constraints and two did not complete the interview related to weight control. The remaining 118 participants were on average 70.8 years old (SD = 7.2, range = 56–89). The majority was female (67.8%), had graduated from primary school (55.9%), had religious beliefs (95.8%), was married (73.7%), and had no job (83.9%). Their average time since OA diagnosis was 55.5 months (SD = 55.0, range = 0–362). The majority of participants had a Kellgren-Lawrence osteoarthritis classification of Stage IV (57.6%). Only 25.4% had body weight in the normal range. About 8% had previously received TKA. For details, see Table 2.

**Table 2:** Participants’ characteristics (*N* = 118)

Characteristic	
Gender	
Male	38 (32.2)
Female	80 (67.8)
Body mass index	
Normal	30 (25.4)
Overweight	38 (32.2)
Mild obesity	35 (29.7)
Moderate obesity	13 (11.0)
Severe obesity	2 (1.7)
Age, years (range = 56–89)	70.8 (7.2)
Education	
Illiterate	12 (10.2)
Primary school	66 (55.9)
Junior high school	18 (15.3)
Senior high school	17 (14.4)
College and above	5 (4.2)
Religious beliefs	
Yes	113 (95.8)
No	5 (4.2)
Marital status	
Single	2 (1.7)
Married	87 (73.7)
Divorced	3 (2.5)
Widowed	26 (22.0)
Number of children (range = 0–7)	3.4 (1.2)
Work status	
Yes	19 (16.1)
No	99 (83.9)
Time since osteoarthritis diagnosis, months (range = 0–362)	55.5 (55.0)
Kellgren-Lawrence classification	
Stage III	50 (42.4)
Stage IV	68 (57.6)
Site of osteoarthritis	
Left knee	39 (33.1)
Right knee	52 (44.1)
Both knees	27 (22.9)
Current treatment for osteoarthritis	
Medication	100 (84.7)
Rehabilitation	3 (2.5)
Medication+ Rehabilitation	15 (12.7)
Previously received total knee arthroplasty	
Yes	8 (6.8)
No	110 (93.2)

Continuous variables are presented by mean and standard deviation, with categorical variables presented by frequency and percentage

### Weight-control strategies

Over half of our participants (*n* = 66, 55.9%) reported having controlled their weight. Their most common weight-control strategies were to control their diet and to combine exercise and diet control together (Table 3). Regarding diet control, the majority of participants tried to decrease the amount of food they ate. As P5 said, “I try to not eat so much. I used to eat all that I could. Now, I’m afraid of eating too much.” Some participants tried to skip dinner or eat less at dinner. As P37 said, “Sometimes, I try to not eat dinner to lose weight. If I feel hungry, I drink water. I think eating dinner is the main reason for gaining weight. For weight control, I eat less at dinner.” Some participants decreased the amount of fat in their diet. In extreme cases, they even became vegetarians. As P7 said, “It’s easy to gain weight during holidays. In the mid-autumn festival, we need to pray using moon cakes. If you eat moon cakes, you will quickly gain weight. Therefore, I replaced moon cakes with fruits or cookies. Similarly, we need to pray in the Chinese New Year. I eat vegetarian pig, chicken, and fish [made from tofu]. I eat almost only vegetables and fruits now. All are very light.”

**Table 3:** Main findings

	Themes
Weight-control strategies	• Control diet (*n* = 51) • Exercise and control diet together (*n* = 15)
Facilitators of weight control	• A desire for good health (*n* = 45) • Wanting to improve walking ability or mobility (*n* = 37) • Perceiving that one had gained weight (*n* = 34) • Wanting to look good (*n* = 11) • Advice from healthcare providers (*n* = 9)
Barriers to weight control	• Perceiving that dietary control was not needed (*n* = 36) • Difficulty controlling appetite (*n* = 10) • Dietary control was difficult (*n* = 7) • Not eating caused physical discomfort (*n* = 7)

In addition, some participants combined diet control and exercise to control their weight. As P119 said, “I climb a mountain every Tuesday. It takes about 4 hours. I always weigh myself every day. I control my weight by dietary control and exercise.” Similarly, P15 said, “I use dietary control and exercise to control my weight. I try to decrease the amount of food I eat and walk half an hour per day.”

### Facilitators of weight control

Participants’ weight control was facilitated by a desire for good health, wanting to improve their walking ability or mobility, perceiving that they had gained weight, wanting to look good, and advice from healthcare providers (Table 3). Regarding the desire for good health, some participants said that losing weight was a way to manage their other chronic diseases. As P46 said, “I have diabetes and hypertension. I need to cut down the amount of rice. To control these conditions, I must eat less and exercise.” However, some participants simply wanted to have good health. As P114 said, “I want to live longer, so I will maintain my good health.”

Some participants expressed having difficulty walking or/and with mobility. As P20 said, “I think that controlling weight might help me with walking. If I wasn’t so fat, it would be easier for me to walk and move around.” Similarly, P65 said, “Being overweight makes it difficult for me to walk. Moreover, it [being overweight] makes me feel short of breath.”

Regarding their perception of having gained weight, some participants monitored their weight by weighing themselves. As P70 said, “I weigh myself every day. If I gain weight, then I eat less.” Some participants used personal methods to guess their weight. As P104 said, “I enjoy eating, especially sweet snacks. That’s why I’m so fat. If I eat more snacks, I know I have gained weight and should eat less for the next couple of days.”

Wanting to look good was a common theme for older women. As P32 said, “I’m very careful about my appearance. I want to look good. You see, I’m already over 80, but I still dye my hair. Being overweight looks bad.” Similarly, P90 said, “I love beautiful things. However, my husband said my taste has become worse. I replied that it was due to my weight; no matter what I wear, it looks bad. Then I start to control my weight.”

As for the facilitator of healthcare providers’ reminder to control weight, P35 said, “My doctor told me that I’m overweight, increasing my risk for surgery. To have TKA, I need to lose some weight.” Similarly, P86 said, “I recently had a health examination. My doctor reminded me that my blood sugar is slightly high, so I need to control my weight and diet.”

### Barriers to weight control

Over 40% of participants said that they did not have any barriers to weight control. The remaining participants described the following barriers to weight control: perceiving that dietary control was not needed, difficulty controlling appetite, dietary control was difficult, and not eating caused physical discomfort (Table 3). Participants’ perceptions of not needing to control their weight was based on three reasoning/perception patterns: 1) they ate regularly, 2) weight was normal or below normal, and 3) they were too old. For the first pattern, P27 said, “I eat the same amount of food [each day] and very regularly. So, I don’t need to control my weight.” For the second pattern, P9 (a mildly obese participant) said, “I tend to be underweight. I don’t need to control my weight.” For the third pattern, P80 said, “I am old and don’t need to care about my appearance. I don’t think about controlling my weight.”

Regarding the barrier of difficulty controlling appetite, P61 said, “When I see food, I can’t control myself. I must eat it. Otherwise, I feel sorry for myself.” Several participants said weight control was difficult. As P74 said, “I don’t know what’s wrong. I eat as other people recommend. However, they lose weight, but I don’t. Sometimes, I even gain more weight. I really don’t know how to control my weight.” Some participants said that not eating caused physical discomfort, e.g., dizziness, stomach pains, hunger, and trouble sleeping. As P20 said, “If I don’t eat, I feel drowsy and dizzy. Sometimes, my hunger prevents me from sleeping.”

## Discussion

Our results contribute to the literature on OA and nutrition by describing weight-control strategies, facilitators of and barriers to weight control among older adults with knee OA who did not feel comfortable undergoing physician-recommended TKA. In addition, our findings provide insights to clinicians caring for older overweight adults waiting for knee-replacement surgeries in countries with waiting lists. These results also contribute to OA and nutrition research in Taiwan, thus expanding related knowledge from western countries. The major strengths of this study are its large sample, particularly for a qualitative study, and the research team’s long engagement with older adults, which allowed them to gain their trust in collecting data on sensitive topics such as weight control and choosing to not undergo knee-replacement surgery. The study’s weaknesses are outlined in the limitations below.

Speaking to weight control, fewer than 60% of participants had controlled their weight even though only about 1/4 weighed in the normal range. Moreover, even though controlling diet was their most common strategy for controlling weight, their methods may have placed them in a malnourished condition. For example, participants said they decreased the amount of food they ate, but they had difficulty describing the exact amount of food and its calories. This eating behavior could result in their eating high-calorie foods or snacking between meals but being unaware of the problem. These adults, like all adults with knee OA, should be educated about healthy eating [9]. A healthy diet emphasizes fruits, vegetables, whole grains, and fat-free or low-fat milk and milk products; it includes lean meats, poultry, fish, beans, eggs, and nuts; is low in saturated fats, trans fats, cholesterol, sodium, and added sugars; and stays within a person’s daily caloric needs [26].

Participants’ perceptions of exercise were vague. Many participants described taking a walk as their exercise. They commonly took a 20- to 30-min walk around a park near their home. However, OA patients are recommended to perform 30 min of moderate-intensity physical activity at least 5 times a week, or 150 min per week [27]. If taking a walk can be counted as moderate-intensity physical activity, each case should be carefully examined.

Our participants described weight control as being facilitated by desiring good health, wanting to improve walking or mobility, perceiving that they had gained weight, wanting to look good, and advice from healthcare providers. Desiring good health as a facilitator of weight control can be explained by the association of overweight and obesity with various chronic diseases [28], such as type 2 diabetes. Our second facilitator of weight control (to improve walking/mobility) is consistent with reports that US patients with knee OA were motivated to lose weight because they wanted to improve their knee symptoms (e.g., pain) [29] and that caregivers of knee OA patients in Taiwan identified knee pain as a main barrier to patients’ walking and mobility [30]. Another facilitator of weight control in our study was a perceived weight gain. Some participants regularly weighed themselves, but some just made a guess. Regularly weighing oneself is important for weight control [9] and should be emphasized in weight-control intervention programs. Our finding that physical appearance was a common motivator for weight loss is consistent with a report of US patients before and after TKA [29]. Finally, we found that weight control was facilitated by advice from healthcare providers. Our finding differs from the conclusion of a systematic review that primary care clinicians in various western countries had personal beliefs about patient adherence and treatment effectiveness that made them reluctant to recommend standard treatment to OA patients [31]. As a result, their advice did not help patients. Thus far, no studies have examined this issue in Asian countries. Future Asian studies should explore the role of health care providers in providing advice to OA patients.

About 45% of our older participants did not describe any barriers to weight control. Since no other studies have reported this finding, no comparison can be made. A majority of our remaining participants perceived that dietary control was not needed. Under this barrier to weight control, participants’ descriptions of not needing weight control was based on three reasoning/perception patterns. First, participants perceived that they ate regularly and did not need to control their weight. However, as people age, their metabolic rate decreases leading to their gaining weight. These patients should be educated on this issue. Second, participants perceived their body weight was normal (even underweight) and did not need to be controlled. Clinicians should not only advise OA patients to weigh themselves regularly, but also emphasize the importance of calculating one’s BMI. Patients must be taught the meaning of BMI rather than only body weight. Third, participants perceived they were too old and did not need to control their weight. These patients need to be informed of medical and technological advances that have increased their life expectancy. Weight control is not only related to appearance but may also promote one’s QOL.

We also found that a barrier to weight control was difficulty controlling appetite, echoing a report that that obese knee OA patients in the UK perceived overconsuming food as a barrier to healthy eating [13]. In addition, our participants described weight loss as difficult to achieve and maintain, as reported for older adults with knee OA in Australia [10]. Another barrier to weight control described by our participants was that not eating caused discomfort, which might be due to low blood sugar.

Some barriers to weight control identified in other studies, e.g., lack of motivation [11], pain in the knee joint [11], pain in other joints [11], problems with calorie counts and food intake record [12], not preparing meals by oneself [12], lack of time for eating [12], desiring high-fat/high-calorie foods [13], and managing mood [13], were not themes in our study. As we described above, one facilitator of our participants’ weight control was to improve walking and mobility [30], which might have been hindered by knee pain. In addition, all our participants were older adults, and most did not work. They might have had more time to prepare and eat food. They also did not feel stressed to consume more food. Interestingly, none of our participants expressed having difficulty counting calories in their food even though they did not know how to do it. This issue suggests a lack of knowledge about calories.

Based on our findings, we have some suggestions for practice and research. Regarding practice, patients with knee OA should first be encouraged to weigh themselves every day and taught about using BMI to examine their body weight. Second, these patients should consult regularly with a dietician, who can teach them about measuring calories, healthy eating, and meal-exchange lists. Third, health care providers should be aware of different patterns of not perceiving a need for weight control and teach these patients the appropriate knowledge to overcome their barriers. Regarding research, we first suggest that the role of health care providers in Asian countries should be explored in providing advice to OA patients. Second, the role of barriers to weight control identified in other studies but not identified by our participants. Should be explored in the weight control of Asian OA patients.

Our findings may be limited by our study using a qualitative descriptive design. Although a descriptive design is useful for understanding participants’ experiences, it is less rigorous than other qualitative designs, such as grounded theory. In addition, older adults who agreed to participate may have had different characteristics from those who rejected participation. Since we are not allowed access to data on nonparticipants, we cannot make comparisons.

## Conclusion

We found that only 25.4% of participants with knee OA had body weight in the normal range even though 55.9% described having controlled their weight. Their most common weight control strategies were to control diet and to exercise and control diet together. Their weight control was facilitated by desiring good health, wanting help with walking or mobility, perceiving that they had gained weight, wanting to look good, and advice from healthcare providers. Common barriers to weight control were perceiving that dietary control was not needed, difficulty controlling appetite, difficulty controlling diet, and not eating caused physical discomfort. Health care providers can integrate these identified facilitators and barriers into a weight-control intervention program to help their patients who did not feel comfortable undergoing physician-recommended TKA to control their weight. Furthermore, the importance of weighing oneself every day, the meaning of BMI, consulting with a dietician regularly to control weight, and providing appropriate knowledge about aging and weight control should also be included in any weight-control intervention program.

## Supplementary information

**Additional file 1.** COREQ (Consolidated criteria for Reporting Qualitative research) Checklist.

## Data Availability

The datasets used and/or analysed during the current study are available from the corresponding author on reasonable request.

## Abbreviations

OA: Osteoarthritis
BMI: Body mass index
QOL: Quality of life
TKA: Total knee arthroplasty
RA: Research assistant
IRB: Institutional Review Board
